# Insight of the Cytotoxicity of the Aggregates of Peptides or Aberrant Proteins: A Meta-Analysis

**DOI:** 10.1371/journal.pone.0095759

**Published:** 2014-04-25

**Authors:** Rong Zhou, Bing Xu

**Affiliations:** Department of Chemistry, Brandeis University, Waltham, Massachusetts, United States of America; Ruhr University Bochum, Germany

## Abstract

Aberrant proteins or peptide aggregates form soluble oligomers or nanofibrils that can cause a wide range of amyloidosis diseases, including Alzheimer's disease (AD). The mechanisms of their cytotoxicity, however, remain controversial and poorly understood, greatly hindering the development of AD drugs. Here we report a comprehensive evaluation of the cytotoxicity of the aggregates by meta-analysis. The analysis indicates that the cytotoxicity of the aggregates converges in a narrower range in the mass concentrations than in the molar concentrations, suggesting that it is the weight of the aggregates rather than the number of the molecules that dictates the cytotoxicity. This new perspective implies that these aggregates are likely to have non-specific interactions with cells to cause cell death. The comparison of several existing theories regarding cellular volumes supports that the aggregates may result in crowding effect and increase the free energy, thus resulting in instability of the cells.

## Introduction

**Figure 1:**
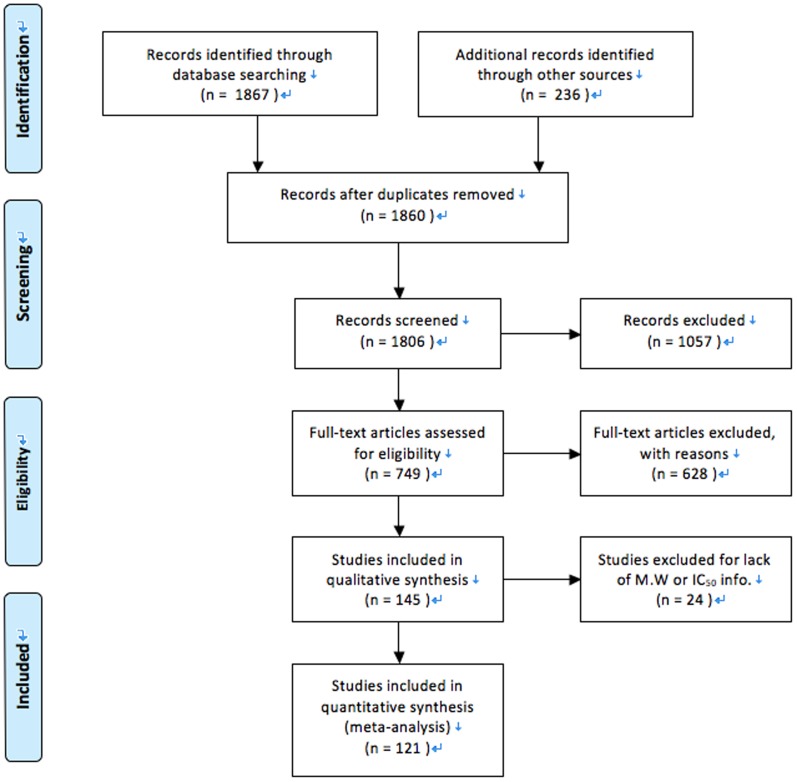
Flow chart of study identification, inclusion, exclusion.

**Figure 2:**
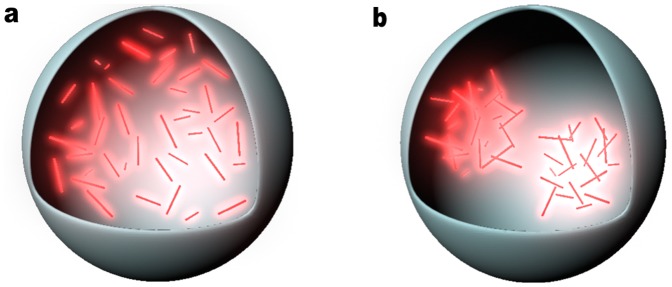
Illustration of the aggregates constrained in a fixed volume and are expressed in different units of concentration. (a) Mole concentration only represents the amounts of individual units in a certain volume. (b) Mass concentration is able to reflect the solid-like properties such as density, which indicates the interaction and accommodates inhomogeneous distribution of the aggregates.

The identification of the nature and mechanisms behind amyloidosis is of great medical value and important because there are around 40 diseases, such as Alzheimer's disease (AD)[2], [3], amyotropic lateral sclerosis (ALS), Huntington's disease, diabetes mellitus, and Parkinson's disease (PD) [4], which are all related to protein misfolding and aggregations. Despite the extensive work done to study the cytotoxicity of various cases of amyloids and aggregates, it still remains challenging to infer a reliable mechanism for explaining the observed phenotypes (e.g., cell death). To address this problem, we analyze the cytotoxicity of aggregates of peptides and aberrant proteins reported in about 628 articles (Figure 1). We use meta-analysis to compare a vast number of cytotoxicity data according to two types of units: µM (10^−6^ mol/L) and mg/mL, the molecular weight, the tested cell lines, and the estimated secondary structure if applicable. The result of the analysis leads to a discovery of the similarity of the cytotoxicity of the aggregates, that is, the IC_50_ values of different aggregates converge in a narrow range according to the mass concentrations (mg/mL). This result implies that the molecular aggregates cause cell death via non-specific interactions (Figure 2).

## Materials and Methods

### Protocols and Eligibility Criteria

The present meta-analysis and systematic review follow the Preferred Reporting Items for Systematic Review and Meta-analysis (PRISMA) statements (Checklist S1). There is no restriction on publication years during the literature search.

### Search Strategy

We have searched the literature that reported cytotoxicity of amyloids and aggregates (as update to June 2013). We receive 1867 hits by using the key words of cytotoxicity, amyloids, or aggregates to search on the Web of Science and Scifinder Scholar. We exclude the non-English papers and the papers on antibody trials and metal nanoparticles (see Supporting Information S1), and we focus our analysis on the rest of articles.

### Selection of Studies

The inclusive criteria are: (1) studies used to validate cytotoxicity measurements (for example, cell number counting and MTT assay); (2) studies with an appropriate analytical design (such as case-control); (3) studies published in English; (4) studies with full-text availability; (5) data were not duplicated in another manuscript.

### Data Extraction

We extract data from the published reports based on certain inclusion and exclusion criteria. For example, the analysis only includes data from the aggregates formed pure compounds (e.g., proteins, polypeptides, or small molecule), and excludes the aggregates formed by a mixture of different molecules. In the case where multiple reports are available for studying the same compound, we chose to extract data from the most up-to-date reports. By using the equation of conversion µM = 10^−6^ mol·L^−1^ = 10^−6^ mg·mL^−1^/(M.W. in g·mol^−1^), we generate the table of cytotoxicity in molar concentration and mass concentration (Table S1).

### Statistical Analysis

In order to reflect the common features of the aggregates, we analyze the reports that focused on Alzheimer's disease (AD) and the reports that focus on diseases or cytotoxicity other than AD (detailed categorization in Table S1). We use the R program (a language and environment for statistical computing and graphic)[5] to analyze the data sets as well as to evaluate the distribution of the data. Since many cytotoxicity results are small values that correspond to the trace amount of aggregates, it is not surprising that the distribution curve skewed to the left (i.e. lower concentration) in the statistic chart. This non-normally distribution excludes the use of t-test here. Thus, we use equivalent tools for non-normally distributed data by choosing one sample Kolmogorov-Smirnov test and Wilcoxon rank sum test to prove that the two data sets are non-parametric, significantly different, and comparable with standard deviations. For all the tests, the nominal level of significance is P-value<2.2e-16.

## Results

**Figure 3:**
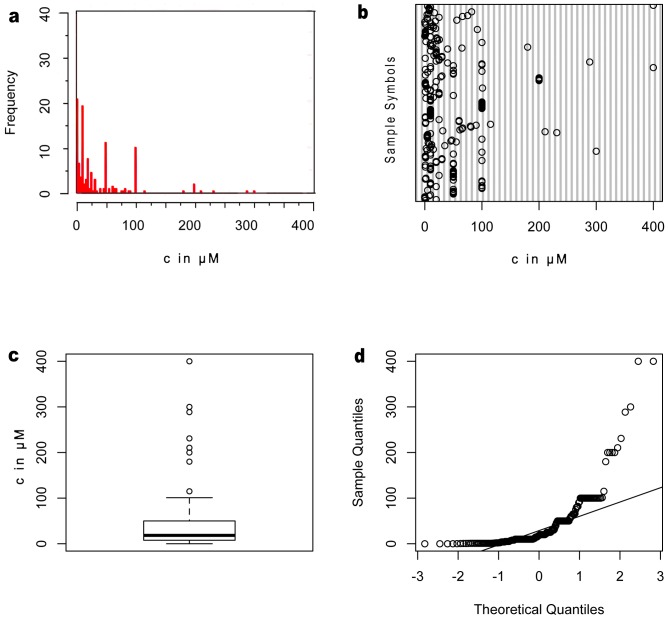
Quantification of cytotoxicity in mole concentration (µM). (a) Histogram, (b) dot plot, (c) box plot and (d) normal Q-Q plot of cytotoxicity data set expressed in µM. While histogram shows how cytotoxicity data spread out in certain concentration range, dot plot gives the global dispersion picture in full concentration range, and box plot brings the mean value as reference. Q-Q plot demonstrates that cytotoxicity in µM as the sample set used in the test, deviates significantly from the normal distribution.

**Figure 4:**
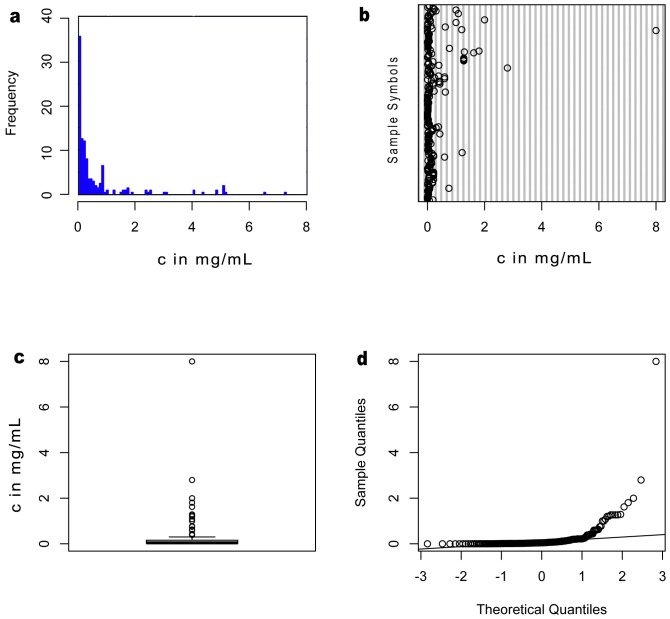
Quantification of cytotoxicity in weight concentration (mg/mL). (a) Histogram, (b) dot plot, (c) box plot, and (d) normal Q-Q plot of cytotoxicity data set expressed in mg/mL. While the histogram, dot plot and box plot imply a narrow distribution of cytotoxicity in mg/mL, Q-Q plot also shows considerable deviation from normal distribution.

When using R program to analyze the data sets, the first step is to examine whether normality applies to their distribution. Thus, we make four plots—histogram, bubble plot, box plot and sample quantities-theoretical quantities (Q-Q) plot–for each data set. The histogram and bubble plot give a global picture about how the data distributed in the full range. The box plot also introduces the median value line to assess the relationship between data distribution and the mean value. As shown in Figure **3a, 3b** and Figure **4a, 4b**, the data in µM exhibit a wider distribution than in mg/mL. Since most of the values of cytotoxicity are quite small, it is not surprising that the distribution skews to the left. The height of the box in Figure **3c** is much longer than that in Figure **4c**, which indicates a highly dispersed distribution and large deviation from the mean value when µM is the unit. To confirm that both data sets are not normally distributed, we use Q-Q plots to compare the data with the normal distribution (i.e., the straight lines in Figure **3d** and **4d**). Both two data sets deviate from the normal distribution to some extent. Thus, we introduce the one sample Kolmogorov-Smirnov test as the equivalent tool for non-normally distributed data to give p-value less than 2.2e-16. This result rejects the hypothesis that two data sets are parametric, which means these data sets are unlikely to have come from any type of probability distribution. Another test we chose is the Wilcoxon rank sum test. It gives p-value less than 2.2e-16 to reject the hypothesis that two data sets are not significantly different. Based on all these results and conclusions, we can compare two data sets with their standard deviations.

Table 1 shows the statistics of the processed results. Obviously, cytotoxicity in mass concentration has a smaller standard deviation value (SD = 0.4831 mg/mL) than that in molar concentration (SD = 1028.7936 µM). The relative standard deviation of data in mass concentration (275.441%) is more than three times lower than that in molar concentration (927.893%). This means that cytotoxicity in mass concentration shows a narrower-sized distribution, hinting at an over-looked common feature of the aggregates. To avoid the vast variability of our data pool, we used the densities of Aβ_1–40_ and Aβ_1–42_, two of the most studied peptide sequences in these reports, as the average density for estimating the possible volume that corresponds to the cytotoxicity in mass concentration. Thus, all the cytotoxicity data would correlate better to the physical volume of the entire aggregate than the average number of molecules uniformly distributed in a fix volume.

**Table 1:** Statistic data of two data sets in mole concentration and mass concentration.

Data Type	Mean	SD	RSD	Maximum	Minimum	Median	Size
Concentration in µM	109.12085	1012.52464	927.893%	15134	1.00E-03	20	223
Concentration in mg/mL	0.24325	0.67001	275.441%	8	1.91E-04	0.0493	223

## Discussion and Conclusion

We find a narrow range of cytotoxicity in mass concentration instead of in molar concentration. Although the conversion of the unit from µM to mg/mL will change the absolute values of the concentrations, it is unlikely to cause the change of the relative distribution of the data. Thus, the narrow distribution in mass concentration hints at an overlooked mechanism. Such parallel comparison provides an alternative perspective for understanding the cytotoxicity of the aggregates. That is, instead of treating aggregates as individual (or monomeric) molecules (which is indicated by mole concentration), it is more suitable to consider the solid-like properties or individual sizes of the aggregates (being reflect more reasonably by the mass concentration). The mass concentration expression depends on the sizes of the aggregates and takes mass, volume, and the interactions of the aggregates with the non-specific molecules into consideration. This result suggests that the volume of aggregates may help elucidate the common behavior of aggregations of the peptides and aberrant proteins. Unquestionably, restricting data to only mass and molar concentrations oversimplifies the situation by not taking into account the differences between different disease-related amyloids. This simple analysis nonetheless provides a new perspective to reconsider the major factors that result in the cytotoxicity of the aggregates of proteins or peptides. One of the implications of this treatment is that the molecular aggregates interact with cell non-specifically to cause the cell death.

Some previous studies had largely focused on certain categories of aggregates. This narrow perspective is likely to overlook certain physical natures that aggregates may exhibit [6]. To find the potential common correlations that can provide new insights, we applied our analytical results to several models: (i) rigid sphere packing model [7], (ii) whole cell simulation model [8], and (iii) crowding effect theory [9], [10]. We first use the rigid sphere surface-packing model [7] to mimic the aggregates of the molecules on the cell membrane surface. However, this model gives a monotonic increasing function of the total volume of aggregation, which corresponds to the change of single particle size. As a result, the function does not converge and fails to show that cytotoxicity of the aggregates fall into a narrow range. Another complication in the analysis of cytotoxicity is that the data sets are concluded from over 800 research papers, excluding studies emphasizing on antibody tests and metal nanoparticles. Therefore the different cell lines and testing methods can vary from each other. The application of the whole cell model [8] with varying mass fractions of proteins only has a subtle influence on the output values of cell stability. Hence it fails to explain the result of the meta-analysis. The whole cell model mainly focused on how all components are incorporated in the cell to establish biological mechanisms. It is understandable that the variables in the model are limited, especially encountering the heterogeneity and the lack of the details of the aggregates.

In comparison, crowding effect theory [4] helps to explain the results from our analysis more effectively. According to crowd effect theory, in the highly crowded interior of a cell, macromolecules physically occupy about 5% to 40% of the total cell volume. Due to the interaction between water and aggregates, an even smaller fraction of the remaining volume is available for other comparable sized molecules to occupy the cell. According to the previous studies [9], when identical globular molecules occupy 30% of a cell's volume, less than 1% of the remaining volume is available to an additional molecule of equal size to insert. This estimation renders a remaining available volume ratio as 0.7% of the total cell volume. Another study [11] shows that the density of a typical aggregation Aβ_1–40_ is 0.49–1.38 g/cm^3^, and 0.72 g/cm^3^ for Aβ_1–42_. Derived from these two results, the total mass of the aggregation that can insert into the cell should be in the range of 2.8–4.9 mg/mL, which is quite compatible with the range of cytotoxicities (0.0002–8 mg/mL) obtained from the experiments in this analysis. This narrow cytotoxicity range, revealed by our meta-analysis, suggesting that crowd effect theory may explain cytotoxicity of the aggregates of aberrant proteins [1].

This meta-analysis combines data from randomized researches for comparing the cytotoxicity in two units, molar concentration and mass concentration. With a total of 223 data sets, a large body of information is available for the evaluation, which allows for some general conclusions to be drawn about the two expressions of cytotoxicity. These researches differ in objectives, methods, and testing cell lines, which may contribute to heterogeneity among the trials. Given such differences, the consistency of cytotoxic range in the results of the meta-analysis is striking. Although neither mass concentration nor mole concentration fully reflects the aggregation states of amyloids (monomer, oligomer or fibril), the use of the mass concentration still helps provide a different perspective than that in mole concentration. The data suggests that after taken molecular interaction into consideration, physical properties, like density and volume of aggregates, play important roles in cytotoxicity effect. The fact that data skewed to the left reflects that cytotoxicity generally tends to correspond to the trace amount of aggregates. This result is also consistent with the conventional unit used in biology. For example, the concentrations of small molecules (e.g., glucose) are in mole concentration, but proteins' (e.g., tubulins) are in mg/mL [12]. This analysis also reveals the extreme cases (e.g., IC_50_ of 0.0002 mg/mL), which may offer new perspective for understanding the cytotoxicity of the aggregates in the cases of outliners.

## Supporting Information

Checklist S1 **PRISMA 2009 Checklist.**
(DOC)

Supporting Information S1 **Computational methods and inclusion/exclusion criteria.**
(DOCX)

Table S1 **Aggregates information from research papers.**
(DOCX)
