# Supplementary material for: Insight of the Cytotoxicity of the Aggregates of Peptides or Aberrant Proteins: A Meta-Analysis
Source: PLoS One. 2014 Apr 25;9(4):e95759. doi: 10.1371/journal.pone.0095759 (PMC4000214; doi:10.1371/journal.pone.0095759)
Supplement: Supporting Information S1 — Computational methods and inclusion/exclusion criteria. (DOCX) [file pone.0095759.s002.docx]

**Analysis of the Cytotoxicity of the Aggregates of Peptides or Aberrant Proteins**

Rong Zhou and Bing Xu*

**SUPPLEMENTARY INFORMATION**

**Computational Method**

We used R software to analyze the data sets’s distribution and features of comparability. The scripts are shown as followings:

>x=c(sample set as concentration in μM)

>y=c(sample set as concentration in mg/mL)

EDA=function(z)

+ {par(mfrow=c(2,2)) #plot as two columns and two rows

+ hist(z); #histogram

+ dotchart(z); #dot plot

+ boxplot(z,horizontal=F); #box plot

+ qqnorm(z);qqline(z) #normal distribution Q-Q plot

+ par(mfrow=c(1,1))}

> r=rank(y, ties.method="random")

> p=rank(x, ties.method="random”)

> ks.test(p,"pnorm")

> ks.test(r,"pnorm")

> wilcox.test(x,y)

**Inclusion/exclusion criteria**

We used computerized literature searching though search engines such as SciFinder® and Web of Science databases for –2013 (search terms: amyloid, aggregate, cytotoxicity) to develop a pool of cytotoxicity samples for analysis. For each research, we extracted lists of compound ID name, molecular weight, cytotoxicity, cell line in use and incubation time when applicable. The inclusion and exclusion criteria were applied. For example, the analysis only includes data from the aggregates formed pure compounds (e.g., proteins, polypeptides, or small molecule), and excludes the aggregates formed by a mixture of different molecules (e.g., clay or metal). In the cases of multiple reports available for studying the same compounds, we chose to extracted data from the most up-to-date reports.

Unit Conversion: μM = 10^-6^ mol∙L^-1^ = 10^-6^ mg∙mL^-1^ / (M.W. in g∙mol^-1^)
