# Supplementary material for: Insight of the Cytotoxicity of the Aggregates of Peptides or Aberrant Proteins: A Meta-Analysis
Source: PLoS One. 2014 Apr 25;9(4):e95759. doi: 10.1371/journal.pone.0095759 (PMC4000214; doi:10.1371/journal.pone.0095759)
Supplement: Table S1 — Aggregates information from research papers. (DOCX) [file pone.0095759.s003.docx]

**Table S1:** Aggregates information from research papers.

| **Aggregates** | **M.W** | **C (μM)** | **c(mg/mL)** | **Cell loss /reduction** | **Incubation Time(h)** | **Cell line** | **PMID** | **Ref** |
| --- | --- | --- | --- | --- | --- | --- | --- | --- |
| **AD Related** | | | | | | | | |
| ASPD | 20000 | 0.0001 | 2 |  |  | NIH-3T3 | 12750461 | ([Hoshi, Sato et al. 2003](#_ENREF_28)) |
| Temporin L | 1640 | 7.5 | 0.0123 |  |  | Hut-78 | 19394305 | ([Mahalka and Kinnunen 2009](#_ENREF_38)) |
| Temporin L | 1640 | 10 | 0.0164 |  |  | K-562 | 19394305 | ([Mahalka and Kinnunen 2009](#_ENREF_38)) |
| Tau590–595 mature fibrils | 713 | 50 | 0.03565 | ~90% |  | PC-12 | 18036611 | ([Pastor, Kuemmerer et al. 2008](#_ENREF_46)) |
| Tau590–595 prefibrillar aggregates | 713 | 50 | 0.03565 | ~70% |  | PC-12 | 18036611 | ([Pastor, Kuemmerer et al. 2008](#_ENREF_46)) |
| Tau590–595 sonicated spec敩ͳऀ | 713 | 50 | 0.03565 | ~55% |  | PC-12 | 18036611 | ([Pastor, Kuemmerer et al. 2008](#_ENREF_46)) |
| Aβ16-21 sonicated species | 723 | 50 | 0.03615 | ~60% |  | PC-12 | 18036611 | ([Pastor, Kuemmerer et al. 2008](#_ENREF_46)) |
| PrP244-249 sonicated species | 738 | 5 | 0.00369 | ~55% |  | PC-12 | 18036611 | ([Pastor, Kuemmerer et al. 2008](#_ENREF_46)) |
| D-PrP245-250 | 738 | 50 | 0.0369 | ~50% |  | PC-12 | 18036611 | ([Pastor, Kuemmerer et al. 2008](#_ENREF_46)) |
| D-CysC98-103 | 727 | 100 | 0.0727 | ~60% |  | PC-12 | 18036611 | ([Pastor, Kuemmerer et al. 2008](#_ENREF_46)) |
| STVIIE prefibrillar aggregates | 7507 | 100 | 0.7507 | ~70% |  | PC-12 | 18036611 | ([Pastor, Kuemmerer et al. 2008](#_ENREF_46)) |
| De novodesigned peptide STVIIE | 660 | 100 | 0.066 |  |  | PC-12 | 18036611 | ([Pastor, Kuemmerer et al. 2008](#_ENREF_46)) |
| Fibrils Aβ1-40 | 4335.89 | 10 | 0.0433589 |  |  | PC-12 | 18059284 | ([Chimon, Shaibat et al. 2007](#_ENREF_13)) |
| NAC(8-18) | 943.53 | 10 | 0.0094353 |  |  | PC-12 | 11461974 | ([Bodles, Guthrie et al. 2001](#_ENREF_7)) |
| NAC(8-16) | 773.43 | 10 | 0.0077343 |  |  | PC-12 | 11461974 | ([Bodles, Guthrie et al. 2001](#_ENREF_7)) |
| hIAPP(20-29) | 1009.08 | 50 | 0.050454 | ~40% |  | pancreatic cell | 19374013 | ([Andrews, Inayathullah et al. 2009](#_ENREF_2)) |
| hIAPP(12-20) | 986 | 50 | 0.0493 | ~35% |  | pancreatic cell | 19374013 | ([Andrews, Inayathullah et al. 2009](#_ENREF_2)) |
| HSV-gB(22-42) | 2095 | 25 | 0.052375 | 45-70% |  | neuronal cell | 10821670 | ([Azizeh, Cribbs et al. 2000](#_ENREF_4)) |
| Aβ1-40 L17λ | 4330 | 50 | 0.2165 |  | 10 | PC-12 | 18078350 | ([Bieschke, Siegel et al. 2008](#_ENREF_5)) |
| Aβ1-40 V18ω | 4330 | 50 | 0.2165 |  | 12 | PC-12 | 18078350 | ([Bieschke, Siegel et al. 2008](#_ENREF_5)) |
| Aβ1-40 F19‑Φ | 4331 | 50 | 0.21655 |  | 60 | PC-12 | 18078350 | ([Bieschke, Siegel et al. 2008](#_ENREF_5)) |
| Aβ1-40 F20‑Φ | 4330 | 50 | 0.2165 |  | 18 | PC-12 | 18078350 | ([Bieschke, Siegel et al. 2008](#_ENREF_5)) |
| Wild type Aβ1-40 | 4330 | 50 | 0.2165 |  | 30 | PC-12 | 18078350 | ([Bieschke, Siegel et al. 2008](#_ENREF_5)) |
| NAC(3-18) fresh | 1413.62 | 10 | 0.0141362 | 64.80% | 24 | PC-12 | 10759841 | ([Bodles, Guthrie et al. 2000](#_ENREF_6)) |
| NAC(3-18) aged | 1413 | 10.00 | 0.01413 | 78.8-49.7% | 24 | PC-12 | 10759841 | ([Bodles, Guthrie et al. 2000](#_ENREF_6)) |
| NAC(1-18s) fresh | 1670.9 | 10 | 0.016709 | 83.1-47.2% | 24 | PC-12 | 10759841 | ([Bodles, Guthrie et al. 2000](#_ENREF_6)) |
| NAC(1-18s) aged | 1670 | 1 | 0.00167 | 80.4-52-38% | 24 | PC-12 | 10759841 | ([Bodles, Guthrie et al. 2000](#_ENREF_6)) |
| NAC(1-174) fresh | 20029.1 | 10 | 0.200291 | 61.2-42.8% | 24 | PC-12 | 10759841 | ([Bodles, Guthrie et al. 2000](#_ENREF_6)) |
| NAC(1-174) aged | 20019 | 1 | 0.020019 | 72.9-42.7-39.1% | 24 | PC-12 | 10759841 | ([Bodles, Guthrie et al. 2000](#_ENREF_6)) |
| AChE586-599 | 1862 | 50 | 0.0931 | ~80-45% | 48 | PC-12 | 12427014 | ([Cottingham, Hollinshead et al. 2002](#_ENREF_16)) |
| Aβ25-35 | 1061.28 | 50 | 0.053064 | ~40 | 48 | PC-12 | 12427014 | ([Cottingham, Hollinshead et al. 2002](#_ENREF_16)) |
| Aβ1-42_1 | 4514 | 20 | 0.09028 | ~58 % | 7D | rat embryonic cortical neurons | 20486703 | ([Fukuda, Matsumoto et al. 2010](#_ENREF_22)) |
| Aβ1-42isoAsp23 | 4514 | 20 | 0.09028 | ~50 % |  | Neuron | 20486703 | ([Fukuda, Matsumoto et al. 2010](#_ENREF_22)) |
| E22G-Aβ1-42 fresh | 4442 | 30 | 0.13326 | ~40 % | 7D+0 | SH-SY5Y | 22475489 | ([Goeransson, Nilsson et al. 2012](#_ENREF_26)) |
| E22G-Aβ1-42 aged | 4442 | 30 | 0.13326 | ~90 % | 7D+30 | SH-SY6Y | 22475489 | ([Goeransson, Nilsson et al. 2012](#_ENREF_26)) |
| dendrimer [G3]-Mor | 12532.3 | 1.5 | 0.01879845 | ~65% |  | CCL-14 | 22206488 | ([Wasiak, Ionov et al. 2012](#_ENREF_66)) |
| EV 1-40 | 4367 | 50 | 0.21835 | ~86% |  | Human SHEP cells | 12684519 | ([Qahwash, Weiland et al. 2003](#_ENREF_49)) |
| Aβ40 | 4325 | 50 | 0.21625 | ~78% |  | Human SHEP cells | 12684519 | ([Qahwash, Weiland et al. 2003](#_ENREF_49)) |
| AβN3(pE)-40 | 4125 | 10 | 0.04125 | 51.00% |  | Hip-pocampal neurones | 12354296 | ([Russo, Violani et al. 2002](#_ENREF_53)) |
| P11-2 | 1600 | 100 | 0.16 | ~60% | 2 | SH-SY5Y | 19393615 | ([Salay, Qi et al. 2009](#_ENREF_55)) |
| Aβ12-28 | 1953 | 10 | 0.01953 | ~50% |  | PC-12 | 12450327 | ([Rabanal, Tusell et al. 2002](#_ENREF_50))FRANCESC RABANAL, et al. J. Peptide Sci. 8: 578–588 (2002) |
| CPDG3 | 16280 | 1 | 0.01628 | ~50% |  | N2a cell | 22206488 | ([Wasiak, Ionov et al. 2012](#_ENREF_66)) |
| CPDG4 | 33702 | 1 | 0.033702 | ~50% |  | N2a cell | 22206488 | ([Wasiak, Ionov et al. 2012](#_ENREF_63)) |
| Aβ42 by p38AF | 4514 | 10 | 0.04514 | _60% |  | M17 cell | 16187214 | ([Zhu, Mei et al. 2005](#_ENREF_89)) |
| Aβ42 | 4514 | 2 | 0.009028 | ~60% |  | Neuron | 16187214 | ([Zhu, Chen et al. 2012](#_ENREF_88)) |
| Aβ23-35 | 1061.2 | 15 | 0.015918 | 85.00% |  | PC-12 | 22540007 | ([Zhou, Li et al. 2011](#_ENREF_87)) |
| HLA20(5-(4-propargylpiperazin-1-yl- methyl)-8-hydroxyquinol) | 281 | 10 | 0.00281 | ~40% |  | SH-SY5Y | 20981484 | ([Zheng, Youdim et al. 2009](#_ENREF_86)) |
| HLA20A | 264 | 50 | 0.0132 | ~35% |  | SH-SY5Y | 20981484 | ([Zheng, Youdim et al. 2009](#_ENREF_86)) |
| M30 | 226 | 25 | 0.00565 |  |  | SH-SY5Y | 20981484 | ([Zheng, Fridkin et al. 2010](#_ENREF_85)) |
| M30D | 297 | 25 | 0.007425 |  |  | SH-SY5Y | 20981484 | ([Zheng, Fridkin et al. 2010](#_ENREF_85)) |
| Aβ1-42 | 4514 | 1 | 0.004514 | ~30% |  | neuron | 11815632 | ([Zhang, McLaughlin et al. 2002](#_ENREF_82)) |
| Aβ40-1 | 4330 | 10 | 0.0433 | ~10% |  | neuron | 11815632 | ([Zhang, McLaughlin et al. 2002](#_ENREF_82)) |
| Cep4b-cAβ1-42 | 4514 | 10 | 0.04514 | ~40% |  | neuron | 11815632 | ([Zhang, McLaughlin et al. 2002](#_ENREF_82)) |
| Aβ40-Cu-FC1 | 4790 | 5 | 0.02395 | ~60% |  | Hela cell | 21437337 | ([Zhang, Chen et al. 2011](#_ENREF_81)) |
| LY294002+Ab+LA | 4514 | 30 | 0.13542 |  |  | neuron | 11602326 | ([Zhang, Xing et al. 2001](#_ENREF_77)) |
| Aβ25-35 | 1060 | 1 | 0.00106 | ~55% |  | NG108-15 | 11602326 | ([Zhang, Xing et al. 2001](#_ENREF_77)) |
| haloperidone | 375.9 | 50 | 0.018795 | 45.00% |  | SH-SY5Y cell | 21523348 | ([Yang and Lung 2011](#_ENREF_68)) |
| risperidone | 410.49 | 100 | 0.041049 | 41.80% |  | SH-SY5Y cell | 21523348 | ([Yang and Lung 2011](#_ENREF_68)) |
| olanzapine | 312 | 100 | 0.0312 | 49.50% |  | SH-SY5Y cell | 21523348 | ([Yang and Lung 2011](#_ENREF_68)) |
| Aβ23-35 | 1061.28 | 40 | 0.0424512 | 49.20% |  | SH-SY5Y cell | 21523348 | ([Yang and Lung 2011](#_ENREF_68)) |
| Aβ40 | 4325 | 20 | 0.0865 | ~55% |  | PC-12 | 21190816 | ([Wang, Lin et al. 2011](#_ENREF_65)) |
| FL-Ab | 5575 | 25 | 0.139375 | ~40% |  | PC12 | 21190816 | ([Wang, Lin et al. 2011](#_ENREF_65)) |
| Aβ25-35 | 1061.28 | 40 | 0.043064 | ~50 | 2 | HAEC | 9262168 | ([Suo, Fang et al. 1997](#_ENREF_61)) |
| Aβ25-36 | 1181.28 | 10 | 0.013064 | ~40 | 48 | HMMs | 22631869 | ([McGuire, Motskin et al. 2012](#_ENREF_42)) |
| Trans2-AMPP | 4500.5 | 1 | 0.045 | 47% |  | C17.2 neural progenitor cells | 22860190 | ([Doran, Anderson et al. 2012](#_ENREF_17)) |
| Trans3-AMPP | 4566.5 | 1 | 0.04566 | 48% |  | C17.2 neural progenitor cells | 22860190 | [Doran, Anderson et al. 2012](#_ENREF_1)) |
| PG-24,25Ab40 | 4332.108 | 1 | 0.0433 | ~30% |  | C17.2 neural progenitor | 22860190 | ([Doran, Anderson et al. 2012](#_ENREF_18)) |
| PG-25,26Ab40 | 4344.219 | 1 | 0.04344 | ~30% |  | C17.2 | 22860190 | ([Doran, Anderson et al. 2012](#_ENREF_18)) |
| PG-26,27Ab40 | 4287.77 | 1 | 0.042870 | ~35% |  | C17.2 | 22860190 | ([Doran, Anderson et al. 2012](#_ENREF_18)) |
| Hemin | 659.90 | 10 | 0.064 | ~50% |  | SH-SY5Y | 22412990 | ([Chuang, Lee et al. 2012](#_ENREF_14)) |
| HL3 | 216 | 92 | 0.019872 | 50% |  | HepG2 | 21783481 | ([Scott, Telpoukhovskaia et al. 2011](#_ENREF_56)) |
| HL4 | 258 | 19 | 0.004902 | 50% |  | HepG2 | 21783481 | ([Scott, Telpoukhovskaia et al. 2011](#_ENREF_56)) |
| cisplatin | 301.1 | 15 | 0.0045165 | 50% |  | HepG2 | 21783481 | ([Scott, Telpoukhovskaia et al. 2011](#_ENREF_56)) |
| Ab31-35 | 600 | 20 | 0.012 | 50% |  | Beta-TC-6 cells | 18566681 | ([Paulsson, Schultz et al. 2008](#_ENREF_47)) |
| Ab34-39 | 600 | 20 | 0.012 | 50% |  | Beta-TC-6 cells | 18566681 | ([Paulsson, Schultz et al. 2008](#_ENREF_47)) |
| **Non-AD Related** | | | | | | | | |
| HypF-N(N-terminal (‘acylphosphatase-like’) domain of  the E. coli HypF protein) | 71347.24 | 1 | 0.07134724 | ~50% |  | NIH-3T3 | 11932737 | ([Bucciantini, Giannoni et al. 2002](#_ENREF_8)) |
| PI3-SH3(phosphatidyl-inositol-3'-kinase) granular type | 9630.67 | 20 | 0.1926134 | ~50% |  | NIH-3T3 | 11932737 | ([Bucciantini, Giannoni et al. 2002](#_ENREF_8)) |
| apomyoglobin mutant W7FW14F | 4218 | 20 | 0.08436 |  |  | NIH-3T3 | 14701846 | ([Sirangelo, Malmo et al. 2004](#_ENREF_59)) |
| PrP106-126 | 1773 | 100 | 0.1773 |  |  | SHSY-5Y | 11461974 | ([Corsaro, Thellung et al. 2003](#_ENREF_15)) |
| PrP106-126 AA | 1801 | 100 | 0.1801 |  |  | SHSY-5Y | 11461974 | ([Corsaro, Thellung et al. 2003](#_ENREF_15)) |
| Alpha-synuclein | 1357.71 | 50 | 0.0678855 |  |  | SH-SY5Y | 22465331 | ([Kingsbury, Laue et al. 2012](#_ENREF_31)) |
| Syrian hamster full-length rPrP23-231 | 23246 | 1 | 0.023246 | < 50% |  | CHO or SKMEL lines | 21625461 | ([Lee, Savtchenko et al. 2011](#_ENREF_36)) |
| hexokinase-B 486 | 108090.78 | 2 | 0.21618156 | > 50% |  | SH-SY5Y | 21249193 | ([Ramshini, Parrini et al. 2011](#_ENREF_52)) |
| Ala 11 | 1454 | 100 | 0.1454 | ~75-58% | 36 | Chinese hamster V79 (male lung, fibroblast) cells  hamster V79 | 14644447 | ([Giri, Ghosh et al. 2003](#_ENREF_25)) |
| Ure2p, soluble oligomer | 119955.04 | 1 | 0.11995504 | ~85-55-38% |  | H-END cells | 16571726 | ([Pieri, Bucciantini et al. 2006](#_ENREF_48)) |
| Ure2p, fibrils αhelix | 119955 | 1 | 0.119955 | ~55-50-40% |  | H-END cells | 16571726 | ([Pieri, Bucciantini et al. 2006](#_ENREF_48)) |
| Ure2p 94-354 | 59932.6 | 10 | 0.599326 | ~100-100-70% |  | H-END cells | 16571726 | ([Pieri, Bucciantini et al. 2006](#_ENREF_48)) |
| ADan | 4046 | 300 | 1.2138 | ~20% | 24 | SH-SY6Y | 18477478 | ([Surolia, Sarkar et al. 2008](#_ENREF_62)) |
| F-NLS-Q42 | 7378 | 4 | 0.029512 | 50.00% |  | PC-12 | 12393802 | ([Yang, Dunlap et al. 2002](#_ENREF_69)) |
| F-NLS-Q20 | 4175 | 15 | 0.062625 | 50.00% |  | PC-12 | 12393802 | ([Yang, Dunlap et al. 2002](#_ENREF_69)) |
| Natural melittin α-helix | 2846 | 2.5 | 0.007115 | 50.00% | 24 | Caco-2 cell | 20025949 | ([Maher, Devocelle et al. 2010](#_ENREF_39)) |
| PA-1 | 2775.42 | 9 | 0.02497878 | ~50% |  | Caco-2 cell | 20025949 | ([Maher, Devocelle et al. 2010](#_ENREF_39)) |
| PA-2 | 2660.29 | 11 | 0.02926319 | ~50% |  | Caco-2 cell | 20025949 | ([Maher, Devocelle et al. 2010](#_ENREF_39)) |
| Core peptide | 790 | 35 | 0.02765 | ~50% |  | Neuronal cell | 20882568 | ([Zhao, Ippolito et al. 2010](#_ENREF_84)) |
| T(L)-peptide | 2195 | 12 | 0.02634 | ~50% |  | Neuronal cell | 20882568 | ([Zhao, Ippolito et al. 2010](#_ENREF_84)) |
| V-peptide | 2182 | 8 | 0.017456 | ~50% |  | Neuronal cell | 20882568 | ([Zhao, Ippolito et al. 2010](#_ENREF_84)) |
| T-peptide | 2179 | 6 | 0.013074 | ~50% |  | Neuronal cell | 20882568 | ([Zhao, Ippolito et al. 2010](#_ENREF_84)) |
| T-peptide | 2179 | 55 | 0.119845 | ~50% |  | HT-22 | 20882568 | ([Zhao, Ippolito et al. 2010](#_ENREF_84)) |
| Arg-Gly-Asp-containing 2 | 1165.17 | 45.7 | 0.053248269 | 50.00% |  | MCF-7 | 19508206 | ([Chen, Chen et al. 2010](#_ENREF_11)) |
| Arg-Gly-Asp-containing 2 | 1165.17 | 47.4 | 0.055221 | 50.00% |  | MDA-MB-453 | 19508206 | ([Chen, Chen et al. 2010](#_ENREF_11)) |
| peptide 1 | 730.74 | 100 | 0.073074 | >50% |  |  | 19508206 | ([Chen, Chen et al. 2010](#_ENREF_11)) |
| cs5931 | 5931 | 5 | 0.029655 | ~50% |  | BEL-7402 | 22440403 | ([Cheng, Wang et al. 2012](#_ENREF_12)) |
| cs5931 | 5931 | 4 | 0.023724 | <50% |  | HCT-8 | 22440403 | ([Cheng, Wang et al. 2012](#_ENREF_12)) |
| cs5931 | 5931 | 4 | 0.023724 | <50% |  | HCT116 | 22440403 | ([Cheng, Wang et al. 2012](#_ENREF_12)) |
| cs5931 | 5931 | 4 | 0.023724 | <50% |  | Hela | 22440403 | ([Cheng, Wang et al. 2012](#_ENREF_12)) |
| cs5931 | 5931 | 5 | 0.029655 | ~50% |  | MCF-7 | 22440403 | ([Cheng, Wang et al. 2012](#_ENREF_12)) |
| cs5931 | 5931 | 10 | 0.05931 | ~50% |  | A549 | 22440403 | ([Cheng, Wang et al. 2012](#_ENREF_12)) |
| Gallidermin | 2069.4 | 231 | 0.4780314 | 50.00% |  | HT29 | 22440403 | ([Cheng, Wang et al. 2012](#_ENREF_12)) |
| Gallidermin | 2064 | 210.5 | 0.434472 | 50.00% |  | Caco-12 | 22440403 | ([Cheng, Wang et al. 2012](#_ENREF_12)) |
| Daunorubicin | 527.52 | 31.5 | 0.01661688 | 50.00% |  | HT29 | 22440403 | ([Cheng, Wang et al. 2012](#_ENREF_12)) |
| Daunorubicin | 527 | 61.1 | 0.0321997 | 50.00% |  | Caco-12 | 22440403 | ([Cheng, Wang et al. 2012](#_ENREF_12)) |
| Magainin I | 2409.85 | 65 | 0.15664025 | 50.00% |  | HT29 | 22440403 | ([Cheng, Wang et al. 2012](#_ENREF_12)) |
| Magainin I | 2409 | 66.3 | 0.1597167 | 50.00% |  | Caco-12 | 22440403 | ([Cheng, Wang et al. 2012](#_ENREF_12)) |
| Magainin II | 2466.9 | 81 | 0.1998189 | 50.00% |  | HT29 | 22440403 | ([Cheng, Wang et al. 2012](#_ENREF_12)) |
| Magainin II | 2466 | 79.9 | 0.1970334 | 50.00% |  | Caco-12 | 22440403 | ([Cheng, Wang et al. 2012](#_ENREF_12)) |
| Nisin A | 3354.07 | 89.9 | 0.301530893 | 50.00% |  | HT29 | 22440403 | ([Cheng, Wang et al. 2012](#_ENREF_12)) |
| Nisin A | 3354 | 115 | 0.38571 | 50.00% |  | Caco-12 | 22440403 | ([Cheng, Wang et al. 2012](#_ENREF_12)) |
| Melittin | 2846 | 1.2 | 0.0034152 | 50.00% |  | HT29 | 22440403 | ([Cheng, Wang et al. 2012](#_ENREF_12)) |
| Melittin | 2846 | 1.8 | 0.0051228 | 50.00% |  | Caco-12 | 22440403 | ([Cheng, Wang et al. 2012](#_ENREF_12)) |
| PrP113-127 | 1097 | 60 | 0.06582 | 70.00% |  | lymphathic cell | 16567397 | ([Murali and Jayakumar 2006](#_ENREF_44)) |
| PrP119-126 | 628 | 60 | 0.03768 | 60.00% |  | lymphathic cell | 16567397 | ([Murali and Jayakumar 2006](#_ENREF_44)) |
| Prp121-127 | 500 | 25 | 0.0125 | 60.00% |  | lymphathic cell | 16567397 | ([Murali and Jayakumar 2006](#_ENREF_44)) |
| parathion | 291.26 | 10 | 0.0029126 | ChAT -20% | 10 D | mixed cells | 15541749 | ([Zurich, Honegger et al. 2004](#_ENREF_91)) |
| chlopyrifos | 350.59 | 10 | 0.0035059 | ChAT 55% | 10 D | mixed cells | 15541749 | ([Zurich, Honegger et al. 2004](#_ENREF_91)) |
| parathion | 291.26 | 10 | 0.0029126 | GAD 20% | 10 D | mixed cells | 15541749 | ([Zurich, Honegger et al. 2004](#_ENREF_91)) |
| chlopyrifos | 350.59 | 10 | 0.0035059 | GAD 32% | 10 D | mixed cells | 15541749 | ([Zurich, Honegger et al. 2004](#_ENREF_91)) |
| parathion | 291.26 | 10 | 0.0029126 | ChAT 12% | 10 D | mixed cells without glial cells | 15541749 | ([Zurich, Honegger et al. 2004](#_ENREF_91)) |
| chlopyrifos | 350.59 | 10 | 0.0035059 | ChAT 10% | 10 D | mixed cells without glial cells | 15541749 | ([Zurich, Honegger et al. 2004](#_ENREF_91)) |
| parathion | 291.26 | 10 | 0.0029126 | GAD 55% | 10 D | mixed cells without glial cells | 15541749 | ([Zurich, Honegger et al. 2004](#_ENREF_91)) |
| chlopyrifos | 350.59 | 10 | 0.0035059 | GAD 10% | 10 D | mixed cells without glial cells | 15541749 | ([Zurich, Honegger et al. 2004](#_ENREF_91)) |
| NP | 139.11 | 100 | 0.013911 | LDH ~40% | 10 D | mixed cells | 15541749 | ([Zurich, Honegger et al. 2004](#_ENREF_91)) |
| TCP | 198.43 | 100 | 0.019843 | LDH ~20% | 10 D | mixed cells | 15541749 | ([Zurich, Honegger et al. 2004](#_ENREF_91)) |
| NP | 139.11 | 100 | 0.013911 | ChAT ~20% | 10 D | mixed cells | 15541749 | ([Zurich, Honegger et al. 2004](#_ENREF_91)) |
| TCP | 198.43 | 100 | 0.019843 | ChAT ~15% | 10 D | mixed cells | 15541749 | ([Zurich, Honegger et al. 2004](#_ENREF_91)) |
| NP | 139.11 | 100 | 0.013911 | GAD ~20% | 10 D | mixed cells | 15541749 | ([Zurich, Honegger et al. 2004](#_ENREF_91)) |
| TCP | 198.43 | 100 | 0.019843 | GAD ~20% | 10 D | mixed cells | 15541749 | ([Zurich, Honegger et al. 2004](#_ENREF_91)) |
| NP | 139.11 | 100 | 0.013911 | GS ~65% | 10 D | mixed cells | 15541749 | ([Zurich, Honegger et al. 2004](#_ENREF_91)) |
| TCP | 198.11 | 100 | 0.019811 | GS ~10% | 10 D | mixed cells | 15541749 | ([Zurich, Honegger et al. 2004](#_ENREF_91)) |
| PbAc | 379.33 | 10 | 0.0037933 | GS ~40% |  | mixed cells | 12237869 | ([Zurich, Eskes et al. 2002](#_ENREF_90)) |
| PbAc | 379.33 | 10 | 0.0037933 | GFAP ~50% |  | mixed cells | 12237869 | ([Zurich, Eskes et al. 2002](#_ENREF_90)) |
| plantaricin A(KSSAYSLQMGATAIKQVKKLFKKWGW) | 2985.57 | 25 | 0.07463925 | ~50% |  | leukemia cells | 16806056 | ([Zhao, Sood et al. 2006](#_ENREF_83)) |
| DT4 | 6189 | 101 | 0.628 | 50.00% |  | Hela cell | 15638538 | ([Zhang, Wang et al. 2005](#_ENREF_80)) |
| DT5 | 13260 | 17.8 | 0.236 | 50.00% |  | Hela cell | 15638538 | ([Zhang, Wang et al. 2005](#_ENREF_80)) |
| DT6 | 34790 | 2.27 | 0.079 | 50.00% |  | Hela cell | 15638538 | ([Zhang, Wang et al. 2005](#_ENREF_80)) |
| DT7 | 64640 | 1.27 | 0.082 | 50.00% |  | Hela cell | 15638538 | ([Zhang, Wang et al. 2005](#_ENREF_80)) |
| DT8 | 113200 | 0.68 | 0.077 | 50.00% |  | Hela cell | 15638538 | ([Zhang, Wang et al. 2005](#_ENREF_80)) |
| PEI | 25000 | 0.72 | 0.018 | 50.00% |  | Hela cell | 15638538 | ([Zhang, Wang et al. 2005](#_ENREF_80)) |
| PLL | 27000 | 1.03 | 0.028 | 50.00% |  | Hela cell | 15638538 | ([Zhang, Wang et al. 2005](#_ENREF_80)) |
| hIAPP(1–37)KCNTATCATQRLANFLVHSSNNFGAILSSTNVGSNTY | 3906 | 20 | 0.07812 | 25.00% |  | INS-1 | 21130765 | ([Zhang, Cheng et al. 2011](#_ENREF_79)) |
| hIAPP(20–29) | 1009.08 | 200 | 0.201816 | 60.00% |  | INS-1 | 21130765 | ([Zhang, Cheng et al. 2011](#_ENREF_79)) |
| hIAPP(17–29) | 2169 | 200 | 0.4338 | 69.00% |  | INS-1 | 21130765 | ([Zhang, Cheng et al. 2011](#_ENREF_79)) |
| pIAPP(20–29) | 1009.08 | 200 | 0.201816 | 84.00% |  | INS-1 | 21130765 | ([Zhang, Cheng et al. 2011](#_ENREF_79)) |
| HIAPP(17–29) | 2169 | 200 | 0.4338 | 60.00% |  | INS-1 | 21130765 | ([Zhang, Cheng et al. 2011](#_ENREF_79)) |
| apo-BLA | 14200 | 28 | 0.4 | 58.00% |  | A549 | 19497410 | ([Zhang, Yang et al. 2009](#_ENREF_78)) |
| B4 | 12000 | 50 | 0.6 | 50.00% |  | RIN-5F cells | 18451519 | ([Zhang, Fujii et al. 2008](#_ENREF_76)) |
| B5 | 5000 | 50 | 0.25 | 50.00% |  | RIN-5F cells | 18451519 | ([Zhang, Fujii et al. 2008](#_ENREF_76)) |
| Ure2p | 119955.04 | 5 | 0.5997752 |  |  | SH-SY5Y/HeLa/HEK-293/MES 23.5 | 22457725 | ([Zhang, Liu et al. 2012](#_ENREF_75)) |
| albebetin | 7784 | 50 | 0.3892 |  |  | cerebellar granular neurons | 16638570 | ([Zamotin, Gharibyan et al. 2006](#_ENREF_73)) |
| Insulin | 11,981 | 1 | 0.011981 |  |  | HEK293/PC12 | 22262644 | ([Zako, Sakono et al. 2012](#_ENREF_72)) |
| beta-2m | 12000 | 10 | 0.12 |  |  | HEK293/PC12 | 22262644 | ([Zako, Sakono et al. 2012](#_ENREF_72)) |
| MPP+ | 170 | 400 | 0.068 | ~40% |  | PC12 | 19540852 | ([Yin, He et al. 2009](#_ENREF_71)) |
| SH3 | 9630.67 | 10 | 0.0963067 | ~60% |  | SH-SY5Y cell | 17412999 | ([Yerbury, Poon et al. 2007](#_ENREF_70)) |
| PAAc-b-PDLLA | 9700 | 288.6 | 2.79942 | 50.00% |  | Hela cell | 22038476 | ([Xue, Huang et al. 2009](#_ENREF_67)) |
| cystatin C | 16000 | 25 | 0.4 |  |  | VSMC | 17963746 | ([Vilhjalmsson, Blondal et al. 2007](#_ENREF_64)) |
| ConA | 7647 | 0.8 | 0.0061176 | >50% |  | LANS cell | 19782769 | ([Vetri, Carrotta et al. 2010](#_ENREF_63)) |
| Alpha-synuclein | 1357.71 | 30 | 0.0407313 | ~45% |  | Dopaminergic cell | 11316809 | [Sung, Kim et al. (2001](#_ENREF_60)) |
| Alpha-synuclein | 1357.71 | 30 | 0.0407313 | ~50% |  | Hippocampal Neurons | 11316809 | [Sung, Kim et al. (2001](#_ENREF_60)) |
| aDrs | 12000 | 16 | 0.192 | ~40% |  | Sf9 insect cell | 19765079 | [Gossler-Schofberger, Hesser et al. (2009](#_ENREF_27)) |
| Tat-ELP1-L12 | 63500 | 20 | 1.27 | ~80% |  | PaCa-2 cell | 19513001 | [Massodi, Thomas et al. (2009](#_ENREF_41)) doi:10.3390/molecules14061999 |
| Tat-ELP1-L12 | 63500 | 20 | 1.27 | ~60% |  | Panc-1 cell | 19513001 | [Massodi, Thomas et al. (2009](#_ENREF_41))[_ENREF_1](#_ENREF_1) |
| Tat-ELP1-L12 | 63500 | 20 | 1.27 | ~60% |  | MCF-7 | 19513001 | [Massodi, Thomas et al. (2009](#_ENREF_41))[_ENREF_1](#_ENREF_1) |
| Tat-ELP1-L12 | 63500 | 20 | 1.27 | ~65% |  | SKOV-3 | 19513001 | [Massodi, Thomas et al. (2009](#_ENREF_41)) doi:10.3390/molecules14062002 |
| BFDMA | 722.4 | 20 | 0.014448 |  |  | COS-7 cell | 22980739 | ([Aytar, Muller et al. 2012](#_ENREF_3)) |
| ToThy | 801 | 65 | 0.052 | 50% |  | SH-SY5Y | 21897988 | ([Simeone, Mangiapia et al. 2011](#_ENREF_58)) |
| ToThy | 801 | 180 | 0.14418 | 50% |  | Hela | 21897988 | ([Simeone, Mangiapia et al. 2011](#_ENREF_58)) |
| klO18/O8 | 135000 | 12 | 1.62 | ~50% |  | HL-1 | 21368874 | ([Sikkink and Ramirez-Alvarado 2010](#_ENREF_57)) |
| AL-09 | 129000 | 10 | 1.29 | ~50% |  | HL-1 | 21368874 | [Sikkink and Ramirez-Alvarado 2010](#_ENREF_32)) |
| AL-12 | 129000 | 14 | 1.806 | ~50% |  | HL-1 | 21368874 | [Sikkink and Ramirez-Alvarado 2010](#_ENREF_32)) |
| K11V-TR | 7700 | 100 | 0.77 | ~50% |  | PC12/Hela/SH-SY5Y | 22403391 | ([Laganowsky, Liu et al. 2012](#_ENREF_33)) |
| Apo SOD1 | 15000 | 2 | 0.030 | ~80% |  | SH-SY5Y | 22558346 | ([Johansson, Vestling et al. 2012](#_ENREF_30)) |
| Compound9 | 625 | 22.31 | 0.013943 | 50% |  | C6 glioma cells | 22558346 | ([Johansson, Vestling et al. 2012](#_ENREF_30)) |
| Compound106 | 639 | 25.43 | 0.016249 | 50% |  | C6 glioma | 22558346 | ([Johansson, Vestling et al. 2012](#_ENREF_30)) |
| Compound11 | 653 | 21.73 | 0.014189 | 50% |  | C6 glioma | 22558346 | ([Johansson, Vestling et al. 2012](#_ENREF_30)) |
| Compound12 | 667 | 20.76 | 0.013846 | 50% |  | C6 glioma | 22558346 | ([Johansson, Vestling et al. 2012](#_ENREF_30)) |
| Compound13 | 695 | 10.06 | 0.00699 | 50% |  | C6 glioma | 22558346 | ([Johansson, Vestling et al. 2012](#_ENREF_30)) |
| ARQ65 | 120000 | 0.0035 | 0.00042 | ~40% |  | SK-N-SH cells | 22366762 | ([Jochum, Ritz et al. 2012](#_ENREF_29)) |
| QCD5-g-CS | 75000.67 | 10.5 | 8 | 50% |  | Buccal mucosal cells | 21300088 | ([Sajomsang, Gonil et al. 2011](#_ENREF_54)) |
| QCD11-g-CS | 81000.24 | 14.7 | 1.2 | 50% |  | Buccal | 21300088 | ([Sajomsang, Gonil et al. 2011](#_ENREF_54)) |
| QCD23-g-CS | 74000.56 | 2.14 | 0.16 | 50% |  | Buccal | 21300088 | ([Sajomsang, Gonil et al. 2011](#_ENREF_54)) |
| Curcumin | 368 | 0.52 | 0.000191 | ~50% |  | L929 fibroblast cell | 20848656 | ([Manju and Sreenivasan 2011](#_ENREF_40)) |
| PVP-curcumin | 3400 | 0.52 | 0.625 | ~50% |  | L929 | 20848656 | ([Manju and Sreenivasan 2011](#_ENREF_40)) |
| β2M | 12000 | 10 | 0.12 | ~50% |  | SH-SY5Y | 21864514 | ([Kong, Cheng et al. 2011](#_ENREF_32)) |
| GC-ADR-5 | 263200 | 0.038 | 0.01 | ~50% |  | HepG2 | 16480840 | ([Park, Cho et al. 2006](#_ENREF_45)) |
| adriamycin | 543 | 18.4 | 0.01 | ~90% |  | Hep2G | 16480840 | ([Park, Cho et al. 2006](#_ENREF_45)) |
| PEI | 25000 | 0.4 | 0.01 | 50% |  | NIH/3T3 | 18023906 | ([Germershaus, Mao et al. 2008](#_ENREF_24)) |
| C50 | 50000 | 20 | 1 | 50% |  | NIH/3T3 | 18023906 | ([Germershaus, Mao et al. 2008](#_ENREF_24)) |
| TMC50 | 450000 | 0.2222 | 0.1 | 50% |  | NIH/3T3 | 18023906 | ([Germershaus, Mao et al. 2008](#_ENREF_24)) |
| PTM50 | 113800 | 0.8787 | 0.1 | 50% |  | NIH/3T3 | 18023906 | ([Germershaus, Mao et al. 2008](#_ENREF_24)) |
| SALeuDA(0.16) | 210000 | 9.5238 | 2.0 | ~50% |  | 3T3 | 22001838 | ([Dutta and Dey 2011](#_ENREF_20)) |
| Helix-z | 1399.6 | 56 | 0.078 | ~60% |  | microglial cell | 21673937 | ([Garcia-Gonzalez and Mas-Oliva 2011](#_ENREF_23)) |
| PEI2500 | 2500 | 5.2 | 0.013 | 50% |  | Hela | 21782238 | ([Endres, Beck-Broichsitter et al. 2011](#_ENREF_21)) |
| PEG500-PCL10k-IPEI2500 | 13000 | 3.69 | 0.048 | 50% |  | Hela | 21782238 | ([Endres, Beck-Broichsitter et al. 2011](#_ENREF_5) |
| aglycon triterpenoids asiatic acid | 720 | 25 | 0.018 | 50％ |  | Hela | 18565534 | ([Rafat, Fong et al. 2008](#_ENREF_51)) |
| madecassic acid | 500 | 66 | 0.033 | 50％ |  | Hela | 18565534 | ([Rafat, Fong et al. 2008](#_ENREF_51)) |
| PHEG-L-pro-L-leu-gly-L-pro-gly-PDM | 106000 | 10 | 1.06 | ~40% |  | Hela | 15661487 | ([Katleen De Winne](http://www.sciencedirect.com/science/article/pii/S0928098704002374) et al. 2005) |
| lysozyme | 14400 | 75 | 1.08 | ~55% |  | SH-SY5Y | 20624399 | ([Mossuto, Dhulesia et al. 2010](#_ENREF_43)) |
| bPEI | 25000 | 4 | 0.1 | ~80% |  | COS-7 | 20857324 | ([Ma, Li et al. 2010](#_ENREF_37)) |
| mPECA_100_ | 29400 | 6.8 | 0.2 | ~50% |  | COS-7 | 20857324 | ([Ma, Li et al. 2010](#_ENREF_37)) |
| mPECA_50_ | 17900 | 16.76 | 0.3 | ~50% |  | COS-7 | 20857324 | ([Ma, Li et al. 2010](#_ENREF_37)) |
| mPECA_25_ | 12200 | 81.96 | 1 | ~50% |  | COS-7 | 20857324 | ([Ma, Li et al. 2010](#_ENREF_37)) |
| APP-CT105 | 10500 | 8 | 0.084 | ~60% |  | PC-12 | 10797560 | ([Lee, Chang et al. 2000](#_ENREF_34)) |
| GST-CT98 | 30000 | 8 | 0.24 | ~40% |  | PC-12 | 10797560 | ([Lee, Chang et al. 2000](#_ENREF_34)) |
| GST-CT46 | 25000 | 8 | 0.2 | ~40% |  | PC-12 | 10797560 | ([Lee, Chang et al. 2000](#_ENREF_34)) |
| MPP+ | 170 | 100 | 0.017 |  |  | Hippocampal Neurons | 15684486 | ([Zhai, Inoue et al. 2005](#_ENREF_74)) |
| a-Syn G68R | 1350 | 10 | 0.01 | ~50% |  | PC-12 | 12873148 | ([Du, Tang et al. 2003](#_ENREF_19)) |
| WtSY | 13000 | 5 | 0.065 | ~50% |  | HTB-148 | 21060871 | ([Buttner, Delay et al. 2010](#_ENREF_9)) |
| MPTP | 173.25 | 400 | 0.0693 | 36% |  | SK-N-SH neuroblastoma | 16212983 | ([Lee, Tsai et al. 2006](#_ENREF_35)) |
| S20G-IAPP | 3900 | 20 | 0.078 | ～60& |  | rat INS-1 beta vell | 22206987 | ([Cao, Tu et al. 2012](#_ENREF_10)) |
| recproIAPP | 8358 | 50 | 0.4179 | 51% |  | Beta-TC-6 cells | 18566681 | ([Paulsson, Schultz et al. 2008](#_ENREF_47)) |
| recN+IAPP | 6224 | 50 | 0.3112 | 54% |  | Beta-TC-6 cells | 18566681 | ([Paulsson, Schultz et al. 2008](#_ENREF_47)) |
| recIAPP | 4918 | 50 | 0.2459 | 28% |  | Beta-TC-6 cells | 18566681 | ([Paulsson, Schultz et al. 2008](#_ENREF_47)) |
| recIAPP+C | 7053 | 50 | 0.3526 | 41% |  | Beta-TC-6 cells | 18566681 | ([Paulsson, Schultz et al. 2008](#_ENREF_47)) |
| Phenylalanine | 165.19 | 15134 | 8 | 50% |  | PC-12 | 22706200 | ([Adler-Abramovich, Vaks et al. 2012](#_ENREF_1)) |

**Note: M. W.** = molecular Weight; **C (μM)** = concentration in unit of micromole; **C(mg/mL)** = Concentration in unit of milligram per milliliter; **Cell loss/reduction** = the percentage of cell death or reduction in the cell experiment for cytotoxicity; **Incubation time (h)** = the incubation time in the cell experiment in unit of hour; **Cell line** = the cell line being used in the cell experiment; **PMID** = PubMed Unique Identifier; **Ref** = Reference.

**References for table S1**

Adler-Abramovich, L., L. Vaks, O. Carny, D. Trudler, A. Magno, A. Caflisch, D. Frenkel and E. Gazit (2012). "Phenylalanine assembly into toxic fibrils suggests amyloid etiology in phenylketonuria." Nature Chemical Biology **8**(8): 701-706.

Andrews, M. E., N. M. Inayathullah, R. Jayakumar and E. J. P. Malar (2009). "Conformational polymorphism and cellular toxicity of IAPP and βAP domains." J. Struct. Biol. **166**(2): 116-125.

Aytar, B. S., J. P. E. Muller, S. Golan, Y. Kondo, Y. Talmon, N. L. Abbott and D. M. Lynn (2012). "Chemical oxidation of a redox-active, ferrocene-containing cationic lipid: Influence on interactions with DNA and characterization in the context of cell transfection." Journal of Colloid and Interface Science **387**: 56-64.

Azizeh, B. Y., D. H. Cribbs, C. W. Cotman and F. M. LaFerla (2000). Fibril formation and neurotoxicity by a herpes simplex virus glycoprotein B fragment with homology to Alzheimer's β-amyloid peptide, Kluwer Academic Publishers.

Bieschke, J., S. J. Siegel, Y. W. Fu and J. W. Kelly (2008). "Alzheimer's A beta peptides containing an isostructural backbone mutation afford distinct aggregate morphologies but analogous cytotoxicity. Evidence for a common low-abundance toxic Structure(s)?" Biochemistry **47**(1): 50-59.

Bodles, A. M., D. J. Guthrie, P. Harriott, P. Campbell and G. B. Irvine (2000). "Toxicity of non-abeta component of Alzheimer's disease amyloid, and N-terminal fragments thereof, correlates to formation of beta-sheet structure and fibrils." Eur J Biochem **267**(8): 2186-2194.

Bodles, A. M., D. J. S. Guthrie, B. Greer and G. B. Irvine (2001). "Identification of the region of non-Aβ component (NAC) of Alzheimer's disease amyloid responsible for its aggregation and toxicity." J. Neurochem. **78**(2): 384-395.

Bucciantini, M., E. Giannoni, F. Chiti, F. Baroni, L. Formigli, J. S. Zurdo, N. Taddei, G. Ramponi, C. M. Dobson and M. Stefani (2002). "Inherent toxicity of aggregates implies a common mechanism for protein misfolding diseases." Nature **416**(6880): 507-511.

Buttner, S., C. Delay, V. Franssens, T. Bammens, D. Ruli, S. Zaunschirm, R. M. de Oliveira, T. F. Outeiro, F. Madeo, L. Buee, M. C. Galas and J. Winderickx (2010). "Synphilin-1 Enhances alpha-Synuclein Aggregation in Yeast and Contributes to Cellular Stress and Cell Death in a Sir2-Dependent Manner." Plos One **5**(10).

Cao, P., L. H. Tu, A. Abedini, O. Levsh, R. Akter, V. Patsalo, A. M. Schmidt and D. P. Raleigh (2012). "Sensitivity of Amyloid Formation by Human Islet Amyloid Polypeptide to Mutations at Residue 20." Journal of Molecular Biology **421**(2-3): 282-295.

Chen, C.-H., M.-K. Chen, K.-C. G. Jeng and F.-D. T. Lung (2010). "Effects of peptidic antagonists of Grb2-SH2 on human breast cancer cells." Protein Pept. Lett. **17**(1): 44-53.

Cheng, L., C. Wang, H. Liu, F. Wang, L. Zheng, J. Zhao, E. Chu and X. Lin (2012). "A Novel Polypeptide Extracted From Ciona savignyi Induces Apoptosis Through a Mitochondrial-Mediated Pathway in Human Colorectal Carcinoma Cells." Clin. Colorectal Cancer **11**(3): 207-214.

Chimon, S., M. A. Shaibat, C. R. Jones, D. C. Calero, B. Aizezi and Y. Ishii (2007). "Evidence of fibril-like beta-sheet structures in a neurotoxic amyloid intermediate of Alzheimer's beta-amyloid." Nature Structural & Molecular Biology **14**(12): 1157-1164.

Chuang, J. Y., C. W. Lee, Y. H. Shih, T. T. Yang, L. Yu and Y. M. Kuo (2012). "Interactions between Amyloid-beta and Hemoglobin: Implications for Amyloid Plaque Formation in Alzheimer's Disease." Plos One **7**(3).

Corsaro, A., S. Thellung, V. Villa, D. R. Principe, D. Paludi, S. Arena, E. Millo, D. Schettini, G. Damonte, A. Aceto, G. Schettini and T. Florio (2003). "Prion protein fragment 106-126 induces a p38 MAP kinase-dependent apoptosis in SH-SY5Y neuroblastoma cells independently from the amyloid fibril formation." Ann. N. Y. Acad. Sci. **1010**(Apoptosis): 610-622.

Cottingham, M. G., M. S. Hollinshead and D. J. T. Vaux (2002). "Amyloid Fibril Formation by a Synthetic Peptide from a Region of Human Acetylcholinesterase that Is Homologous to the Alzheimer's Amyloid-β Peptide." Biochemistry **41**(46): 13539-13547.

Doran, T. M., E. A. Anderson, S. E. Latchney, L. A. Opanashuk and B. L. Nilsson (2012). "An Azobenzene Photoswitch Sheds Light on Turn Nucleation in Amyloid-beta Self-Assembly." Acs Chemical Neuroscience **3**(3): 211-220.

Doran, T. M., E. A. Anderson, S. E. Latchney, L. A. Opanashuk and B. L. Nilsson (2012). "Turn Nucleation Perturbs Amyloid beta Self-Assembly and Cytotoxicity." Journal of Molecular Biology **421**(2-3): 315-328.

Du, H. N., L. Tang, X. Y. Luo, H. T. Li, J. Hu, J. W. Zhou and H. Y. Hu (2003). "A peptide motif consisting of glycine, alanine, and valine is required for the fibrillization and cytotoxicity of human alpha-synuclein." Biochemistry **42**(29): 8870-8878.

Dutta, P. and J. Dey (2011). "Drug solubilization by amino acid based polymeric nanoparticles: Characterization and biocompatibility studies." International Journal of Pharmaceutics **421**(2): 353-363.

Endres, T. K., M. Beck-Broichsitter, O. Samsonova, T. Renette and T. H. Kissel (2011). "Self-assembled biodegradable amphiphilic PEG-PCL-lPEI triblock copolymers at the borderline between micelles and nanoparticles designed for drug and gene delivery." Biomaterials **32**(30): 7721-7731.

Fukuda, T., E. Matsumoto, S. Onogi and Y. Miura (2010). "Aggregation of Alzheimer Amyloid beta Peptide (1-42) on the Multivalent Sulfonated Sugar Interface." Bioconjugate Chemistry **21**(6): 1079-1086.

Garcia-Gonzalez, V. and J. Mas-Oliva (2011). "Amyloidogenic Properties of a D/N Mutated 12 Amino Acid Fragment of the C-Terminal Domain of the Cholesteryl-Ester Transfer Protein (CETP)." International Journal of Molecular Sciences **12**(3): 2019-2035.

Germershaus, O., S. R. Mao, J. Sitterberg, U. Bakowsky and T. Kissel (2008). "Gene delivery using chitosan, trimethyl chitosan or polyethylenglycol-graft-trimethyl chitosan block copolymers: Establishment of structure-activity relationships in vitro." Journal of Controlled Release **125**(2): 145-154.

Giri, K., U. Ghosh, N. P. Bhattacharyya and S. Basak (2003). "Caspase 8 mediated apoptotic cell death induced by β-sheet forming polyalanine peptides." FEBS Lett. **555**(2): 380-384.

Goeransson, A.-L., K. P. R. Nilsson, K. Kaagedal and A.-C. Brorsson (2012). "Identification of distinct physiochemical properties of toxic prefibrillar species formed by Aβ peptide variants." Biochem. Biophys. Res. Commun. **420**(4): 895-900.

Gossler-Schofberger, R., G. Hesser, M. Muik, C. Wechselberger and A. Jilek (2009). "An orphan dermaseptin from frog skin reversibly assembles to amyloid-like aggregates in a pH-dependent fashion." Febs Journal **276**(20): 5849-5859.

Hoshi, M., M. Sato, S. Matsumoto, A. Noguchi, K. Yasutake, N. Yoshida and K. Sato (2003). "Spherical aggregates of β-amyloid (amylospheroid) show high neurotoxicity and activate tau protein kinase I/glycogen synthase kinase-3β." Proc. Natl. Acad. Sci. U. S. A. **100**(11): 6370-6375.

Jochum, T., M. E. Ritz, C. Schuster, S. F. Funderburk, K. Jehle, K. Schmitz, F. Brinkmann, M. Hirtz, D. Moss and A. C. B. Cato (2012). "Toxic and non-toxic aggregates from the SBMA and normal forms of androgen receptor have distinct oligomeric structures." Biochimica Et Biophysica Acta-Molecular Basis of Disease **1822**(6): 1070-1078.

Johansson, A. S., M. Vestling, P. Zetterstrom, L. Lang, L. Leinartaite, M. Karlstrom, J. Danielsson, S. L. Marklund and M. Oliveberg (2012). "Cytotoxicity of Superoxide Dismutase 1 in Cultured Cells Is Linked to Zn2+ Chelation." Plos One **7**(4).

Kingsbury, J. S., T. M. Laue, S. F. Chase and L. H. Connors (2012). "Detection of high-molecular-weight amyloid serum protein complexes using biological on-line tracer sedimentation." Anal. Biochem. **425**(2): 151-156.

Kong, F. L., W. Cheng, J. Chen and Y. Liang (2011). "D-Ribose glycates beta(2)-microglobulin to form aggregates with high cytotoxicity through a ROS-mediated pathway." Chemico-Biological Interactions **194**(1): 69-78.

Laganowsky, A., C. Liu, M. R. Sawaya, J. P. Whitelegge, J. Park, M. L. Zhao, A. Pensalfini, A. B. Soriaga, M. Landau, P. K. Teng, D. Cascio, C. Glabe and D. Eisenberg (2012). "Atomic View of a Toxic Amyloid Small Oligomer." Science **335**(6073): 1228-1231.

Lee, J. P., K. A. Chang, H. S. Kim, S. S. Kim, S. J. Jeong and Y. H. Suh (2000). "APP carboxyl-terminal fragment without or with A beta domain equally induces cytotoxicity in differentiated PC12 cells and cortical neurons." Journal of Neuroscience Research **60**(4): 565-570.

Lee, W. S., W. J. Tsai, P. H. Yeh, B. L. Wei and W. F. Chiou (2006). "Divergent role of calcium on A beta- and MPTP-induced cell death in SK-N-SH neuroblastoma." Life Sciences **78**(11): 1268-1275.

Lee, Y. J., R. Savtchenko, V. G. Ostapchenko, N. Makarava and I. V. Baskakov (2011). "Molecular structure of amyloid fibrils controls the relationship between fibrillar size and toxicity." PLoS One **6**(5): e20244.

Ma, M., F. Li, X. H. Liu, Z. F. Yuan, F. J. Chen and R. X. Zhuo (2010). "Self-assembled micellar aggregates based monomethoxyl poly(ethylene glycol)-b-poly(epsilon-caprolactone)-b-poly(aminoethyl methacrylate) triblock copolymers as efficient gene delivery vectors." Journal of Materials Science-Materials in Medicine **21**(10): 2817-2825.

Mahalka, A. K. and P. K. J. Kinnunen (2009). "Binding of amphipathic α-helical antimicrobial peptides to lipid membranes: Lessons from temporins B and L." Biochim. Biophys. Acta, Biomembr. **1788**(8): 1600-1609.

Maher, S., M. Devocelle, S. Ryan, S. McClean and D. J. Brayden (2010). "Impact of amino acid replacements on in vitro permeation enhancement and cytotoxicity of the intestinal absorption promoter, melittin." Int J Pharm **387**(1-2): 154-160.

Manju, S. and K. Sreenivasan (2011). "Synthesis and Characterization of a Cytotoxic Cationic Polyvinylpyrrolidone-Curcumin Conjugate." Journal of Pharmaceutical Sciences **100**(2): 504-511.

Massodi, I., E. Thomas and D. Raucher (2009). "Application of Thermally Responsive Elastin-like Polypeptide Fused to a Lactoferrin-derived Peptide for Treatment of Pancreatic Cancer." Molecules **14**(6): 1999-2015.

McGuire, E. K., M. Motskin, B. Bolognesi, S. D. Bergin, T. P. J. Knowles, J. Skepper, L. M. Luheshi, D. W. McComb, C. M. Dobson and A. E. Porter (2012). "Selenium-Enhanced Electron Microscopic Imaging of Different Aggregate Forms of a Segment of the Amyloid beta Peptide in Cells." Acs Nano **6**(6): 4740-4747.

Mossuto, M. F., A. Dhulesia, G. Devlin, E. Frare, J. R. Kumita, P. P. de Laureto, M. Dumoulin, A. Fontana, C. M. Dobson and X. Salvatella (2010). "The Non-Core Regions of Human Lysozyme Amyloid Fibrils Influence Cytotoxicity." Journal of Molecular Biology **402**(5): 783-796.

Murali, J. and R. Jayakumar (2006). "Lymphocyte toxicity of prion fragments." J. Biochem. **139**(3): 329-338.

Park, J. H., Y. W. Cho, Y. J. Son, K. Kim, H. Chung, S. Y. Jeong, K. Choi, C. R. Park, R. W. Park, I. S. Kim and I. C. Kwon (2006). "Preparation and characterization of self-assembled nanoparticles based on glycol chitosan bearing adriamycin." Colloid and Polymer Science **284**(7): 763-770.

Pastor, M. T., N. Kuemmerer, V. Schubert, A. Esteras-Chopo, C. G. Dotti, d. l. P. M. Lopez and L. Serrano (2008). "Amyloid Toxicity Is Independent of Polypeptide Sequence, Length and Chirality." J. Mol. Biol. **375**(3): 695-707.

Paulsson, J. F., S. Schultz, M. Kohler, I. Leibiger, P. O. Berggren and G. T. Westermark (2008). "Real-Time Monitoring of Apoptosis by Caspase-3-Like Protease Induced FRET Reduction Triggered by Amyloid Aggregation." Experimental Diabetes Research.

Pieri, L., M. Bucciantini, D. Nosi, L. Formigli, J. Savistchenko, R. Melki and M. Stefani (2006). "The Yeast Prion Ure2p Native-like Assemblies Are Toxic to Mammalian Cells Regardless of Their Aggregation State." J. Biol. Chem. **281**(22): 15337-15344.

Qahwash, I., K. L. Weiland, Y. Lu, R. W. Sarver, R. F. Kletzien and R. Yan (2003). "Identification of a mutant amyloid peptide that predominantly forms neurotoxic protofibrillar aggregates." J Biol Chem **278**(25): 23187-23195.

Rabanal, F., J. M. Tusell, L. Sastre, M. R. Quintero, M. Cruz, D. Grillo, M. Pons, F. Albericio, J. Serratosa and E. Giralt (2002). "Structural, kinetic and cytotoxicity aspects of 12-28 beta-amyloid protein fragment: A reappraisal." Journal of Peptide Science **8**(10): 578-588.

Rafat, M., K. W. Fong, A. Goldsipe, B. C. Stephenson, S. T. Coradetti, G. Sambandan, A. J. Sinskey and C. Rha (2008). "Association (micellization) and partitioning of aglycon triterpenoids." Journal of Colloid and Interface Science **325**(2): 324-330.

Ramshini, H., C. Parrini, A. Relini, M. Zampagni, B. Mannini, A. Pesce, A. A. Saboury, M. Nemat-Gorgani and F. Chiti (2011). "Large proteins have a great tendency to aggregate but a low propensity to form amyloid fibrils." PLoS One **6**(1): e16075.

Russo, C., E. Violani, S. Salis, V. Venezia, V. Dolcini, G. Damonte, U. Benatti, C. D'Arrigo, E. Patrone, P. Carlo and G. Schettini (2002). "Pyroglutamate-modified amyloid β-peptides - AβN3(pE) -strongly affect cultured neuron and astrocyte survival." J. Neurochem. **82**(6): 1480-1489.

Sajomsang, W., P. Gonil, U. R. Ruktanonchai, N. Pimpha, I. Sramala, O. Nuchuchua, S. Saesoo, S. Chaleawlert-umpon and S. Puttipipatkhachorn (2011). "Self-aggregates formation and mucoadhesive property of water-soluble beta-cyclodextrin grafted with chitosan." International Journal of Biological Macromolecules **48**(4): 589-595.

Salay, L. C., W. Qi, B. Keshet, L. K. Tamm and E. J. Fernandez (2009). "Membrane interactions of a self-assembling model peptide that mimics the self-association, structure and toxicity of Aβ(1-40)." Biochim. Biophys. Acta, Biomembr. **1788**(9): 1714-1721.

Scott, L. E., M. Telpoukhovskaia, C. Rodriguez-Rodriguez, M. Merkel, M. L. Bowen, B. D. G. Page, D. E. Green, T. Storr, F. Thomas, D. D. Allen, P. R. Lockman, B. O. Patrick, M. J. Adam and C. Orvig (2011). "N-Aryl-substituted 3-(beta-D-glucopyranosyloxy)-2-methyl-4(1H)-pyridinones as agents for Alzheimer's therapy." Chemical Science **2**(4): 642-648.

Sikkink, L. A. and M. Ramirez-Alvarado (2010). "Cytotoxicity of amyloidogenic immunoglobulin light chains in cell culture." Cell Death & Disease **1**.

Simeone, L., G. Mangiapia, C. Irace, A. Di Pascale, A. Colonna, O. Ortona, L. De Napoli, D. Montesarchio and L. Paduano (2011). "Nucleolipid nanovectors as molecular carriers for potential applications in drug delivery." Molecular Biosystems **7**(11): 3075-3086.

Sirangelo, I., C. Malmo, C. Iannuzzi, A. Mezzogiorno, M. R. Bianco, M. Papa and G. Irace (2004). "Fibrillogenesis and Cytotoxic Activity of the Amyloid-forming Apomyoglobin Mutant W7FW14F." J. Biol. Chem. **279**(13): 13183-13189.

Sung, J. Y., J. Kim, S. R. Paik, J. H. Park, Y. S. Ahn and K. C. Chung (2001). "Induction of neuronal cell death by Rab5A-dependent endocytosis of alpha-synuclein." Journal of Biological Chemistry **276**(29): 27441-27448.

Suo, Z. M., C. H. Fang, F. Crawford and M. Mullan (1997). "Superoxide free radical and intracellular calcium mediate A beta(1-42) induced endothelial toxicity." Brain Research **762**(1-2): 144-152.

Surolia, I., D. P. Sarkar and S. Sinha (2008). "Form and dimensions of aggregates dictate cytotoxicities of Danish dementia peptides." Biochem. Biophys. Res. Commun. **372**(1): 62-66.

Vetri, V., R. Carrotta, P. Picone, M. Di Carlo and V. Militello (2010). "Concanavalin A aggregation and toxicity on cell cultures." Biochimica Et Biophysica Acta-Proteins and Proteomics **1804**(1): 173-183.

Vilhjalmsson, D. T., H. Blondal and F. R. Thormodsson (2007). "Solubilized cystatin C amyloid is cytotoxic to cultured human cerebrovascular smooth muscle cells." Experimental and Molecular Pathology **83**(3): 357-360.

Wang, S. S. S., M. S. Lin, S. L. Chen, Y. Chang, R. C. Ruaan and W. Y. Chen (2011). "Using isothermal titration calorimetry to real-time monitor the heat of metabolism: A case study using PC12 cells and A beta(1-40)." Colloids and Surfaces B-Biointerfaces **83**(2): 307-312.

Wasiak, T., M. Ionov, K. Nieznanski, H. Nieznanska, O. Klementieva, M. Granell, J. Cladera, J.-P. Majoral, A. M. Caminade and B. Klajnert (2012). "Phosphorus Dendrimers Affect Alzheimer's (Aβ1-28) Peptide and MAP-Tau Protein Aggregation." Mol. Pharmaceutics **9**(3): 458-469.

Xue, Y. N., Z. Z. Huang, J. T. Zhang, M. Liu, M. Zhang, S. W. Huang and R. X. Zhuo (2009). "Synthesis and self-assembly of amphiphilic poly(acrylic acid-b-DL-lactide) to form micelles for pH-responsive drug delivery." Polymer **50**(15): 3706-3713.

Yang, M. C. and F. W. Lung (2011). "Neuroprotection of paliperidone on SH-SY5Y cells against beta-amyloid peptide(25-35), N-methyl-4-phenylpyridinium ion, and hydrogen peroxide-induced cell death." Psychopharmacology **217**(3): 397-410.

Yang, W., J. R. Dunlap, R. B. Andrews and R. Wetzel (2002). "Aggregated polyglutamine peptides delivered to nuclei are toxic to mammalian cells." Hum. Mol. Genet. **11**(23): 2905-2917.

Yerbury, J. J., S. Poon, S. Meehan, B. Thompson, J. R. Kumita, C. M. Dobson and M. R. Wilson (2007). "The extracellular chaperone clusterin influences amyloid formation and toxicity by interacting with prefibrillar structures." Faseb Journal **21**(10): 2312-2322.

Yin, W. L., J. Q. He, B. Hu, Z. S. Jiang and X. Q. Tang (2009). "Hydrogen sulfide inhibits MPP(+)-induced apoptosis in PC12 cells." Life Sciences **85**(7-8): 269-275.

Zako, T., M. Sakono, T. Kobayashi, K. Sorgjerd, K. P. R. Nilsson, P. Hammarstrom, M. Lindgren and M. Maeda (2012). "Cell Interaction Study of Amyloid by Using Luminescent Conjugated Polythiophene: Implication that Amyloid Cytotoxicity Is Correlated with Prolonged Cellular Binding." Chembiochem **13**(3): 358-363.

Zamotin, V., A. Gharibyan, N. V. Gibanova, M. A. Lavrikova, D. A. Dolgikh, M. P. Kirpichnikov, I. A. Kostanyan and L. A. Morozova-Roche (2006). "Cytotoxicity of albebetin oligomers depends on cross-beta-sheet formation." Febs Letters **580**(10): 2451-2457.

Zhai, H. F., T. Inoue, M. Moriyama, T. Esumi, Y. Mitsumoto and Y. Fukuyama (2005). "Neuroprotective effects of 2,5-diaryl-3,4-dimethyltetrahydrofuran neolignans." Biological & Pharmaceutical Bulletin **28**(2): 289-293.

Zhang, C., Y. G. Liu, J. Gilthorpe and J. R. C. van der Maarel (2012). "MRP14 (S100A9) Protein Interacts with Alzheimer Beta-Amyloid Peptide and Induces Its Fibrillization." Plos One **7**(3).

Zhang, D. D., I. Fujii, C. Z. Lin, K. Ito, H. S. Guan, J. E. Zhao, M. Shinohara and M. Matsukura (2008). "The stimulatory activities of polysaccharide compounds derived from algae extracts on insulin secretion in vitro." Biological & Pharmaceutical Bulletin **31**(5): 921-924.

Zhang, L., G. Q. Xing, J. L. Barker, Y. Chang, D. Maric, W. Ma, B. s. Li and D. R. Rubinow (2001). "α-lipoic acid protects rat cortical neurons against cell death induced by amyloid and hydrogen peroxide through the Akt signalling pathway." Neurosci. Lett. **312**(3): 125-128.

Zhang, M., F. Yang, J. Chen, C. Y. Zheng and Y. Liang (2009). "Cytotoxic aggregates of alpha-lactalbumin induced by unsaturated fatty acid induce apoptosis in tumor cells." Chemico-Biological Interactions **180**(2): 131-142.

Zhang, X., B. A. Cheng, H. Gong, C. Z. Li, H. Chen, L. Zheng and K. Huang (2011). "Porcine islet amyloid polypeptide fragments are refractory to amyloid formation." Febs Letters **585**(1): 71-77.

Zhang, X. Q., X. L. Wang, S. W. Huang, R. X. Zhuo, Z. L. Liu, H. Q. Mao and K. W. Leong (2005). "In vitro gene delivery using polyamidoamine dendrimers with a trimesyl core." Biomacromolecules **6**(1): 341-350.

Zhang, Y., L. Y. Chen, W. X. Yin, J. Yin, S. B. Zhang and C. L. Liu (2011). "The chelation targeting metal-A beta 40 aggregates may lead to formation of A beta 40 oligomers." Dalton Transactions **40**(18): 4830-4833.

Zhang, Y., R. McLaughlin, C. Goodyer and A. LeBlanc (2002). "Selective cytotoxicity of intracellular amyloid beta peptide(1-42) through p53 and Bax in cultured primary human neurons." Journal of Cell Biology **156**(3): 519-529.

Zhao, H., R. Sood, A. Jutila, S. Bose, G. Fimland, J. Nissen-Meyer and P. K. J. Kinnunen (2006). "Interaction of the antimicrobial peptide pheromone Plantaricin A with model membranes: Implications for a novel mechanism of action." Biochimica Et Biophysica Acta-Biomembranes **1758**(9): 1461-1474.

Zhao, K., G. Ippolito, L. Wang, V. Price, M. H. Kim, G. Cornwell, S. Fulenchek, G. A. Breen, W. J. Goux and S. R. D'Mello (2010). "Neuron-selective toxicity of Tau peptide in a cell culture model of neurodegenerative tauopathy: Essential role for aggregation in neurotoxicity." J. Neurosci. Res. **88**(15): 3399-3413.

Zheng, H. L., M. Fridkin and M. B. H. Youdim (2010). "Site-Activated Chelators Derived from Anti-Parkinson Drug Rasagiline as a Potential Safer and More Effective Approach to the Treatment of Alzheimer's Disease." Neurochemical Research **35**(12): 2117-2123.

Zheng, H. L., M. B. H. Youdim and M. Fridkin (2009). "Site-Activated Multifunctional Chelator with Acetylcholinesterase and Neuroprotective-Neurorestorative Moieties for Alzheimer's Therapy." Journal of Medicinal Chemistry **52**(14): 4095-4098.

Zhou, Y. Q., W. X. Li, L. Xu and L. Y. Chen (2011). "In Salvia miltiorrhiza, phenolic acids possess protective properties against amyloid beta-induced cytotoxicity, and tanshinones act as acetylcholinesterase inhibitors." Environmental Toxicology and Pharmacology **31**(3): 443-452.

Zhu, X. L., C. Chen, D. Ye, D. N. Guan, L. Ye, J. L. Jin, H. Zhao, Y. T. Chen, Z. Y. Wang, X. Wang and Y. Xu (2012). "Diammonium Glycyrrhizinate Upregulates PGC-1 alpha and Protects against A beta(1-42)-Induced Neurotoxicity." Plos One **7**(4).

Zhu, X. W., M. Mei, H. G. Lee, Y. Wang, J. H. Han, G. Perry and M. A. Smith (2005). "P38 activation mediates amyloid-beta cytotoxicity." Neurochemical Research **30**(6-7): 791-796.

Zurich, M. G., C. Eskes, P. Honegger, M. Berode and F. Monnet-Tschudi (2002). "Maturation-dependent neurotoxicity of lead acetate in vitro: Implication of glial reactions." Journal of Neuroscience Research **70**(1): 108-116.

Zurich, M. G., P. Honegger, B. Schilter, L. G. Costa and F. Monnet-Tschudi (2004). "Involvement of glial cells in the neurotoxicity of parathion and chlorpyrifos." Toxicology and Applied Pharmacology **201**(2): 97-104.
